# Supplementary material for: Hydrophobic Paper-Based SERS Sensor Using Gold Nanoparticles Arranged on Graphene Oxide Flakes
Source: Sensors (Basel). 2019 Dec 11;19(24):5471. doi: 10.3390/s19245471 (PMC6960698; doi:10.3390/s19245471)
Supplement: Supplementary file 1 [file sensors-19-05471-s001.pdf]

Supplementary Materials:

Hydrophobic Paper-Based SERS Sensor by Gold Nanoparticles Arranged on Graphene Oxide Flakes

Dong-Jin Lee[1] and Dae Yu Kim[1,2*]

[1] Inha Research Institute for Aerospace Medicine, Inha University, Incheon 22212, Republic of Korea
[2] Department of Electrical Engineering, College of Engineering, Inha University, Incheon 22212, Republic of Korea
* Correspondence: dyukim@inha.ac.kr; Tel.: +82-032-860-7394

1. Contact angle measurements

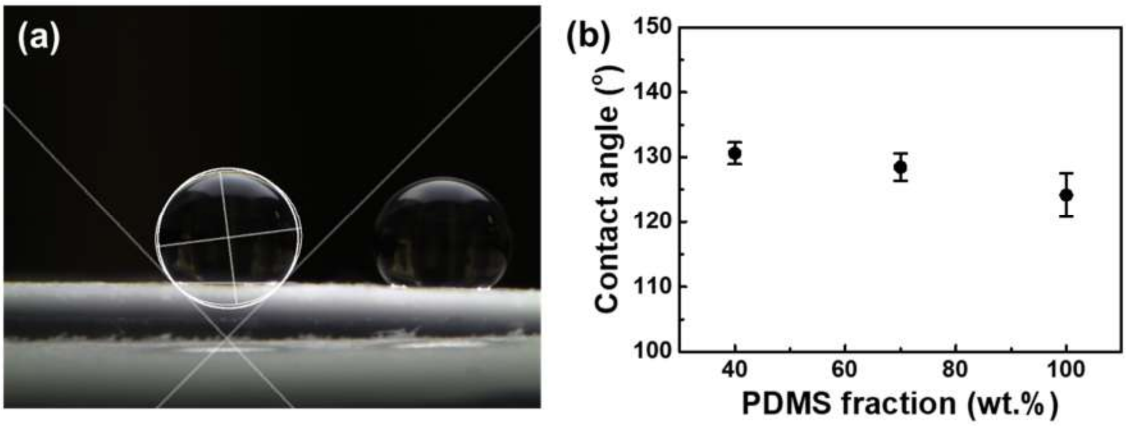

Figure 1. (a) The representative shape of water droplets on h-paper substrate. (b) The contact angle measurements of h-paper substrates with different PDMS fraction.

2. Au NPs counting

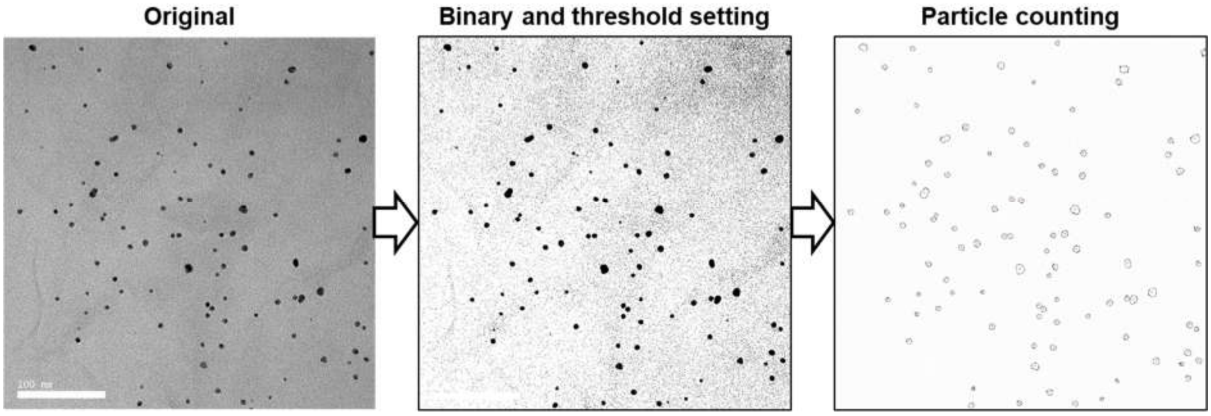

Figure 2. Particle counting process. Original image is converted to binary image, and a threshold range is manually set. The Au NPs are automatically counted using ImageJ software.

3. Reproducibility

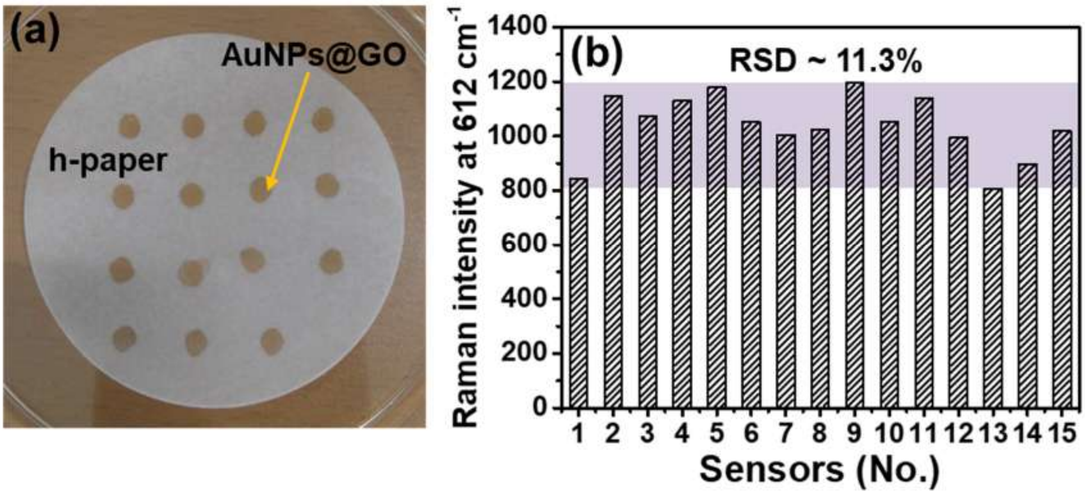

Figure S1. (a) Photographic image of the proposed AuNPs@GO/h-paper SERS substrates. (b) Raman intensity at 612 cm^{-1} of 10^{-3} M R6G measured by 15 Au NPs@GO/h-paper SERS substrates. The relative standard deviation is about 11.3%.

4. Reliability

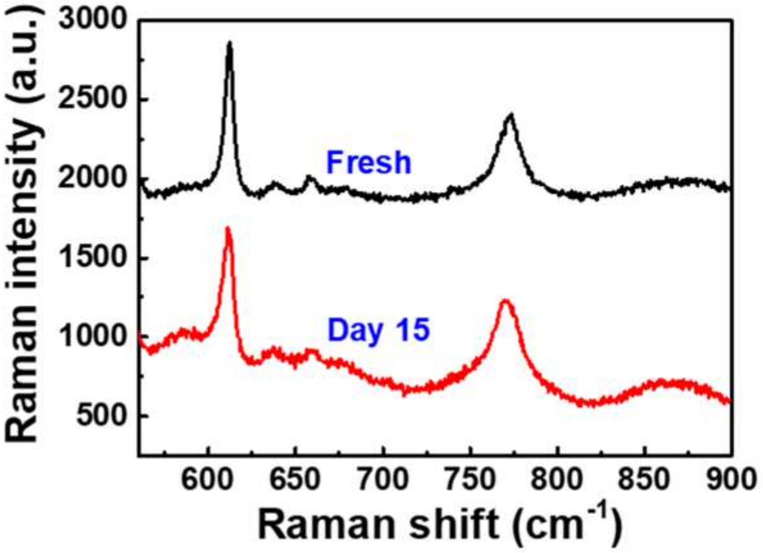

Figure 4. Comparison of the Raman intensity of AuNPs@GO/h-paper SERS substrates that kept for 0 and 15 days.

5. Raman characteristic peak of R6G molecule

Table 1. Raman characteristic peak of R6G molecule.

Peak wavenumber (cm^{-1})	Assignment
613	In plane xanthene ring deformations; Out of plane xanthene ring deformations
775	Out of plane C-H bend; In plane xanthene ring deformations
1184	In plane xanthene ring deformations; In plane C-H bend; In plane N-H bend
1312	In plane xanthene ring breath; In plane N-H bend; CH_2 wag
1364	Xanthene ring stretch; In plane C-H bend
1512	Xanthene ring stretch; C-N stretch; C-H bend; N-H bend
1577	Xanthene ring stretch; in plane N-H bend
1651	Xanthene ring stretch; in plane C-H bend
